# Exploring Women’s Experiences of Maternity Service Delivery in Regional Tasmania: A Descriptive Qualitative Study

**DOI:** 10.3390/healthcare10101883

**Published:** 2022-09-27

**Authors:** Sally Hargreaves, Sarah Young, Sarah J. Prior, Jennifer Ayton

**Affiliations:** 1 Tasmanian Health Service, Latrobe, TAS 7000, Australia; 2 Tasmanian School of Medicine, University of Tasmania, Newnham, TAS 7248, Australia; 3 Tasmanian School of Medicine, University of Tasmania, Burnie, TAS 7320, Australia; 4 Tasmanian School of Medicine, University of Tasmania, Hobart, TAS 7000, Australia

**Keywords:** mothers, continuity, communication, care, maternity services

## Abstract

The objective of this study is to explore and understand the experiences of women who receive antenatal, birthing, and postnatal care from an integrated maternity services model in a regional area in Tasmania, Australia. This descriptive qualitative study included semi-structured, one-on-one interviews with 14 mothers aged >18 years, who were living in a regional area of Tasmania and had accessed maternity health services. Thematic analysis revealed three key themes: (i) talking about me, (ii) is this normal? and (iii) care practices. Overall, women cited mostly negative experiences from a poorly implemented fragmented service. These experiences included feelings of isolation, frustration over receiving conflicting advice, feeling ignored, and minimal to no continuity of care. In contrast, women also experienced the euphoric feelings of birth, immense support, guidance, and encouragement. Regional women’s experiences of maternity care may be improved if health services work towards place-based continuity of care models. These models should be informed by the local women’s experiences and needs in order to achieve better communication, reduce feelings of isolation, and promote positive breastfeeding experiences.

## 1. Introduction

The receipt of equitable maternity care is an essential part of a pregnant woman and her family’s lives. The World Health Organization (WHO) states that each stage of maternal health should be a positive experience, ensuring that women and their babies reach their full potential for health and wellbeing [1]. The care women and their families receive, or have previously received, can shape their emotional and physical wellbeing from pregnancy to motherhood [2]. Negative experiences such as moral judgement, and lack of access, impact women’s mental health in the immediate postpartum, their partner relationship, and their attachment to their baby [2]. Negative experiences have also made some women question their choices about further pregnancies and where and with whom their pregnancy care will be [2]. Conversely, women who have had positive and empowering birth experiences often find that these experiences enrich their emotional selves and empower them as they transition to motherhood [2].

The Australian National Maternity Services plan [3] recommends that pregnant women have better access to models that promote continuity of care, shared decision making, and person-centered care throughout their pregnancy and post-natal journey. Continuity of care is continuous care from the same care provider(s) across the patient’s journey. It allows development of relationships between women and carer [4]. Globally, continuity of care is a well-recognised and well-known model of maternity care [5]. It has been highlighted in the literature as a key component of a positive experience in pregnancy, which increases the importance of maternity services providing access to continuity for women thereby promoting woman-centred care [6]. Continuity of care provides women with improved maternal and neonatal outcomes based on a relationship built on trust and compassion [7]. Further, these models lead to a reduction in negative experiences that women may face throughout their journey to motherhood. The health and wellbeing of mothers and babies can be improved by reducing feelings of isolation, improving communication between mother and carer, and reducing inconsistencies across their care [4]. An evaluation of an integrated maternity care model utilised in the Netherlands suggests that continuity of care, client-centredness and collaborative relationships contribute to a higher quality experience for a woman and her family during pregnancy [8]. In addition to this, care women receive should be safe, effective, patient-centred, timely and efficient as well as accessible; the experience and outcome for the woman as an individual is defined as what is significant and beneficial to her [9].

While hospital-based maternity care is the most common care model in Australia, there remains a need for context-appropriate maternity care models in remote and regional areas. Despite this, many maternity hospitals across Australia have now closed their rural and regional care facilities, leading to centralisation of services in larger metropolitan areas [10]. These structural changes may contribute to models of care that do not meet the unique maternity healthcare needs of regional families and in turn impact on the experiences of women and their families throughout their maternity care experience [10]. Russell et al. [11], highlighted that women living in rural areas are likely to experience difficulties accessing maternity care which can impede their ability to make informed decisions about their pregnancy journey. Difficulties include displacement of families from their remote and regional communities, as pregnant women are required to travel large distances to access safe maternity care [12]. Women who live in remote areas have cited increased financial and emotional burdens as a result as well as limited options of care providers and choice of birth setting [13]. The absence of birthing services in rural areas also impacts First Nations women, many noting the loss of cultural and spiritual aspects of birthing [10]. Homer [6], suggests that the lack of continuity of care models, seen in in regional and remote maternity services, leads to poor communication and conflicting advice impacting on the experiences of the women. Although global recommendations for models of maternity care are valuable, standardised global strategies are inappropriate and do not take into consideration local availability of resources, finances, geography, population demographics and capacity [14].

The aim of this study is to explore and understand women’s experiences of receiving maternity care in a regional Australian context.

## 2. Materials and Methods

This descriptive qualitative study underpinned by postpositivism, used a pragmatic approach [15] to purposefully sample women, aged >18 years, of any parity, who were up to six weeks post birth and living in northwest regional Tasmania to share their experiences of receiving maternity care. The postpositivist perspective supports the documentation of the women’s reality and places the researcher in a pseudo-objective-neutral role. From this stance, the lived experiences of women who received maternity care in a regional area can be described [15]. The Extended Care Midwifery (ECM) service, which visits women in their home, supported the recruitment by providing potential participants with written and verbal introductory information about the study at the completion of their home visit. If the participants agreed to be part of the research, the midwives requested their first name and contact number and provided the participants with an information sheet about the research project with contact details of the researchers. The research team were not involved in the clinical care of the participants. Ethics approval was obtained by the University of Tasmania Human Research and Ethics Committee H0024365 (H-77593).

### 2.1. Setting

This research took place in a northwest regional area of Tasmania classified as very remote to a large rural town [16] with a combined population of approximately 114,000 people [17]. Maternity services for this area are provided by two health care sectors: a private hospital (26 beds inclusive of birthing and postnatal beds) provider delivering inpatient and birthing services to women and a public hospital provider offering antenatal and postnatal, in-home and outpatient, services to women in the area. For women living in the more remote areas, local and travelling midwives, provide antenatal and postnatal care services.

### 2.2. Data Collection

A total of 26 women expressed interest by providing their name and phone number to the Extended Care Midwife (ECM). Women were then contacted by text message by SH to initiate participation in the study. A second text message was sent two days later if there was no response to the initial text. The participant was then removed from the list after a further two days. Of those contacted, 14 agreed to a one-to-one semi-structured phone interview conducted in the participant’s home at a time convenient for each participant. Verbal consent was obtained at the time of the phone interview. All interviews were conducted by SH, (midwife), audio recorded, and then transcribed verbatim by SH. A pilot interview was conducted prior to commencing the study to ensure the interview questions were appropriate and flowed suitably [15]. Minimal changes to the initial questions were made but included additions of structured questions such as, demographic information, parity, how long they had lived in the area, their support network, and to discuss their experience of antenatal, birthing, and postnatal care in their local service. After each interview, the research team debriefed and discussed the interview, and field notes were documented. No repeat interviews with participants were necessary, there were opportunities at the end of the interviews to seek clarification.

### 2.3. Data Analysis

In keeping with a descriptive qualitative study design all data, including field notes, were combined, and analysed thematically, using a predefined coding framework derived from the aim of the study [15]. Our focus was on how women described their lived experiences, consequently, the pre-defined codes were quality, safety, and experiences. All participants were given a pseudonym. Three female research team members (with backgrounds in social sciences, midwifery, and public health) first independently read and reread the data before meeting to discuss and agree on the pre-defined coding framework [18]. SH then coded the data to the agreed predefined codes, meeting with the other team members after each of the four coding iterations, to discuss the development of the emergent sub-themes, expanding and reducing the themes until the final three (Is this Normal; Talking about me, not with me; the Luck of the draw) emerged. Any discrepancies were resolved during each of the four iterations through discussion as a way to ensure trustworthiness and rigor [19]. QSR International NVivo version 11 was used to collate and manage the data [20].

## 3. Results

Of the 14 women interviewed, six were first time mothers. For all women in this study, pregnancy, birth, and post birth care were felt to be a significant personal event. The women understood and valued the importance of sharing their stories because they wanted to be heard and have their experiences documented. They hoped that this would provide insight into their journeys and that their experiences could change some aspects of the service delivery and design in regional areas. All women cited the lack of continuity of care throughout their pregnancy, birthing, and postnatal care. All women also wanted to ensure the often isolating, frustrating, distressing, and empowering experiences of their birth and postnatal care were heard. The data from this study were organised into three main themes as described below.

### 3.1. Themes

#### 3.1.1. Theme 1: Is This Normal?

This theme relates to women’s divergent experiences of maternity care. Most women felt that their maternity care was different to what they expected, citing isolation and confusion. This led women to question what was normal. Many women emphasised the uncertainty surrounding their expectations of care including the information they received during the antenatal period, inclusivity in the birth suite and the inpatient support, or lack of, surrounding their breastfeeding experiences. Anise discussed her confusion when she was on the postnatal ward as she expected the same care she received with her previous births and therefore felt isolated and uncertain. She said:

*To be honest I was ignored. I didn’t get breakfast at all. It wasn’t even acknowledged that I hadn’t eaten. I like they did my standard checks and stuff. But apart from that I didn’t really no one checked in on me no one did anything they kind of just ignored me I don’t know if it was because it was baby number five, or, you know, as I just sort of expected though I knew whether it was busy I don’t know. But I must say that that was the one thing that I was quite upset about was you know, I hadn’t been given breakfast I’d literally just had a baby…..* *it just wasn’t acknowledged at all that, I’d expressed a concern about him [baby] not accepting one breast, he’d accept one breast but the other one, he wouldn’t, and that concern wasn’t really even looked into, it was sort of maybe you could just try a different hold and that was it*

(Anise 5th baby)

The sense of isolation and confusion about what to expect from care providers and the service is suggestive of the lack of connection and continuity across the continuum of care. Throughout the study, some women compared their current experience in the local area to their previous experiences elsewhere, which had built their expectations about their maternity care and compared their care throughout their interview. Some women highlighted their frustrations of the fragmented care and closure to services in the local area. For example, Anise (5th baby) talked of how pleasant her experience had been prior to the integration of the maternity services. She went on to describe some aspects of her care within the current maternity services as *horrendous.* Similarly, others talked of going to another hospital and area for their next baby thinking that *care must be better elsewhere* (Pepperberry 1st baby). Generally, these conflicting experiences lead some women to explore other options such as alternative maternity care for their next pregnancy.

Overall, women described how frequently they felt *left alone*, often ignored and receiving *no help*. The feeling of isolation were more prominent when describing their experiences during birthing and their immediate postnatal care. Women often felt confused as it did not appear to be what the women understood and knew should occur. Women felt that there was an assumption about who needed care, that *if it isn’t your first baby, you have remembered what to do* (Aster 4th baby). Overall, most women at times felt *fobbed off* during their care. Rosemary noticed it more so whilst an inpatient, she mentioned *no one came near me* on the postnatal ward. Similarly, Tulip (2nd baby) described feeling *pretty much ignored* and how unpleasant this felt, citing that she was better off at home. For those having their first babies, their sense of isolation was heightened citing how they felt lost.

Many women reflected on how they felt anxious about feeding, describing how they wanted reassurance relating to breastfeeding. Ivy (1st baby) talked of feeling *traumatised*, and she recounted that she was having trouble feeding and no one was listening to her cries for help. The feelings of being left alone and not having the appropriate help were overwhelming for most of the women in the study. Daphne (1st baby) cited that her location and the resultant lack of resources and options available to her were tangible, especially postnatally in the community.

Generally, the data suggest that all women experienced poor communication, a lack of accountability, and minimal engagement, and thus questioned the normality of their care and the responsibility of staff throughout their experience. Women talked of feeling like they were another statistic in the system as they navigated the service model that exists within this regional Tasmanian area. Many cited that they could not determine whether this was routine (normal) care or whether they were being treated differently.

#### 3.1.2. Theme 2: Talking about Me Not with Me

From the data, women felt there were many occasions when the communication pertaining to their care and the care of their baby was inadequate. Mothers often cited how midwives and doctors appeared to have poor communication and interpersonal skills, deepening their sense of isolation. As one mother said, *I guess that was the thing that stood out for me that you know I’d try to say it a couple of times, but it was sort of dismissed* (Aster 4th baby). During the antenatal care period, women felt that they needed a *frank discussion* about what to expect and prepare them for birthing and motherhood. All women described the overwhelming number of pamphlets provided during their antenatal visits and women discussed their experience during their antenatal care as task orientated and felt the experience to be a tick box situation. Rose (1st baby) mentioned, *they kind of gave us the pamphlets and didn’t really talk to us like it wasn’t a conversation and then they just ticked it off in the book because we’ve been through it*. Conversely, some women felt that the midwives truly invested and appreciated their journey towards motherhood. Almond (1st baby) cited that the midwives thoroughly explained to her about pregnancy and what to expect when giving birth and breastfeeding.

All women mentioned at some point during their experiences of maternity care, that they had feelings of being ignored or not listened to. The women expressed that these moments resulted in feeling disregarded and helpless whilst requesting help or seeking advice. One mother, unwell during her pregnancy, explained how her experience was stressful especially during the COVID 19 pandemic:

*…and so I’d had a cold and I had a chest infection was being treated so that, because I had those symptoms so wasn’t allowed to attend any appointments, getting pushed back and pushed back and that kind of felt like I got to 38 weeks and I was like I haven’t seen a doctor like I need to be seeing a doctor, what is happening …that was stressful because I was missing appointments and I just let like I was being left behind, why doesn’t anyone care, why doesn’t anyone care that I’m really unwell while pregnant*

(Daphne 1st baby)

It was clear that women felt left out of the care process. Though many talked of actively seeking out help and advice, the maternity care system did not appear to enable active partnerships between the mother and care provider.

Despite this, women described seeking reassurance from staff about their care. However, the sense of helplessness intertwined with being ignored was overwhelming for some. Aster (4th baby) recounted how she saw midwives sitting at the nursing station whilst she was in labour with her fourth baby. She reflected that all she wanted was someone to come in and ask if *I needed anything, as you would think they would have heard me making noises*. Similarly, one woman said that it would be nice if they just listened because, *honestly I was kind of angry when I left, you know the fact I was pretty much ignored was not pleasant.* (Aster 4th baby). Pepperberry (1st baby), described how she felt, that there was no one to help her when she kept pressing the bell to ask for a midwife to check her and her baby:

*I asked to go home just because there was no help there, yeah, they let me go. I realised there was, there was no point really staying in there when I am asking for help and they’re not helping me.*

These experiences suggest women felt trapped in uncertainty when they felt ignored. Having a baby is a significant change in a woman’s life [2] and their sense of being dismissed or not listened to was hard for them to comprehend. This was often compounded by a feeling of being objectified as a task within their maternity care system. The experience of disconnect between women, midwives, and doctors were apparent in the women’s experiences of maternity care.

Most women felt that they were being *talked about* rather than being spoken to, during their labour. This made women feel that they were not important in the decision-making process, and in turn, that what they thought, was not important. Many women felt frustrated because staff were talking in the corner of the room, women found this concerning that their care was being decided for them and felt excluded. Whilst labouring, Pepperberry (1st baby) said she could hear staff talking about her. She went on to explain that *their conversations [about me] behind me, really annoyed me*. Magnolia (1st baby) was in disbelief that the communication surrounding her birth was so poor, and she described her support person trying to listen to what the midwife and doctor were discussing in the room so he could understand what was happening. Conversely, there were some women who highlighted their positive experiences during labour. The support from the midwives throughout their birthing experience made a significant difference. For example, Frangipani (2nd baby) was empowered and euphoric that she had experienced the birth she wanted. She described her midwife as having an *amazing calmness* and how they spoke words of support to her, which made a positive difference to her experience.

Women’s experiences appeared to be dependent on certain aspects of their maternity care. For example, women in this study all received extended care midwifery (ECM) once discharged from the birth hospital. Women spoke highly of this service as this service offered breastfeeding support and postnatal care to women and their babies in their homes. Some women described a sense of relief because the ECM midwives provided support and advice that met their individual needs. All women described how caring and knowledgeable the ECM midwives were. One woman talked of how happy she was breastfeeding her baby after the ECM had visited, citing that it was the best feed he [baby] had since being discharged. (Anise 5th baby). Overall, for many women in this study their postnatal care with ECM was felt to be more personalised and citing how relieved they felt when they saw the same midwife.

#### 3.1.3. Theme 3: Luck of the Draw

The final theme refers to how the women in this study perceived continuity of care and how women experienced poor continuity throughout their experiences. Women noticed the lack of continuity and saw how this impacted of some aspects of their care. Women frequently talked about seeing different midwives. Rosemary (1st baby) noted, it would have been better to see only one midwife in order to build a relationship and promote understanding of her story and journey. Almond (1st baby) cited that throughout her care she saw many midwives, she said *don’t expect anything different from the public system, it is what you get, that is, you won’t see anyone the same*. Similarly, Tulip (2nd baby) cited how she would have preferred to see one midwife consistently through her care. However, she did note that even though she saw different midwives *all the midwives made me feel comfortable and I always felt supported*.

The fragmented maternity services model that exists in regional Tasmania has contributed to the minimal continuity women have experienced throughout their maternity care. Women discussed the inconsistent advice they had received, and regardless of their circumstances, wanted reassurance from staff surrounding their care. Having the same midwife would alleviate this. For example, Lavender (3rd baby) explained the frustrations of needing to repeat her story to staff members in the birthing suite. She described the lack of continuity as *the luck of the draw who you see*. Similarly, the postnatal experience on the ward for women was viewed as difficult. Olive (1st baby) discussed how she felt she saw *15 different midwives* throughout her time in the hospital over a period of three days. Conversely, women cited the ECM services as mitigating the confusion and frustration experienced in the hospital. Violet (2nd baby) said the ECM midwives were:

*Really friendly and understanding, and they are patient, they explained stuff yeah. It made me feel quite good actually, to be able talk to them and just not feel like I’m being judged or anything like that. Yeah, I feel quite good about it, yeah.*

Women did not discuss a relationship between continuity of care and poor staffing levels during their antenatal care. In the post-natal ward, however, women felt that the lack of continuity was a result of poor staffing levels. Over half the women described how as a result their care was incomplete, citing the busyness of the ward and lack of midwives on duty. Magnolia (1st baby) talked of hearing staff within the ward talking in the corridor and one *barking orders*. Anise reflected feeling sorry for the staff, *you really noticed that they were under the pump*. The experiences suggest that women fell victim to staffing models that exist within healthcare surrounding workforce.

Many women spoke about being placed on a labour timeline when giving birth. Almond was unsure as to why this was the case during her birth experience. Magnolia had a similar experience; she was told that her contractions were not effective enough. She also felt the interruptions and the inability to have the time to birth her baby affected her progress:

*Because my labour had stalled by the time they said an hour, and then another half hour and then had another half an hour because they had to get theatre staff in. At the time, when you sort of put that many timelines on someone, I don’t think they can progress in the zone.*

(Magnolia 1st baby)

Such timelines were indicative of a medicalised model within the maternity services [21]. The data suggest that women unbeknownst to them were put on these timelines to potentially suit the needs of the hospital and not necessarily for the women. All women discussed breastfeeding in their experiences relating to their maternity care. Whilst on the ward, women discussed their requests for help and the desire to have reassurance with breastfeeding. Ivy (1st baby) described her experience as traumatic: *They all said my latch was fine, my nipples were so cracked, and it was painful, but they just forced me to feed even when I told them it was excruciating*.

Other women felt their requests for help with breastfeeding were not met. Breastfeeding advice was inconsistent and confusing depending on who provided care. As Olive (1st baby) said, *I had had one person tell me to do this and then someone else told me to do that and then I saw I was getting really upset*. For women having their second babies, including Frangipani, their experiences were similar. Frangipani cited that she felt the advice surrounding her breastfeeding was not aligned with her needs, and it made her feel like she was doing a *bad job*. Conversely, some women shared their sense of relief when they got home after the ECM midwives’ visits and talked about how *they [midwives] were amazing, absolutely amazing and they were both lactation consultants as well* (Tulip 2nd baby). The inconsistent care received was highlighted in the data, most prominent in regard to breastfeeding experiences on the hospital ward, suggesting that women struggled to comprehend the advice provided to them.

Lastly, we asked all women in the study what advice they would give a hypothetical friend if they were pregnant in the same local area. Some women said that they would advise their friend *to go elsewhere* for a better experience. Overall, women described a lack of trust in the maternity care system, suggesting that other women should be aware of their options, and educate themselves.

## 4. Discussion

This study sought to explore and document women’s experiences of maternity care in regional Tasmania. The findings provide insight into the experiences women had throughout their maternity care through three overarching themes highlighting the complexity of the experiences of women in maternity care. Childbirth is one of the most significant life experiences for a woman and her family. These experiences are important as they can have long term effects on women’s postnatal experience and general health [2]. Sharing their experiences of maternity care allowed the women in this study to verbalise their experiences which were at times emotionally upsetting, but also empowering. Whilst we recognise most women had negative overall experiences, women described positive aspects of their care.

A common thread between the identified themes was that women’s experiences were divergent due to a lack of care continuity especially in a fragmented service model. Women highlighted positive experiences when the principles of continuity were mentioned by way of their care with the ECM midwives, empowering births with a known midwife and when they had supportive staff and received consistent advice. Women highlighted their negative experiences when they felt isolated, confused and when their expectations of what they perceived as normal care practices were not met.

Continuity of care are known to improve outcomes for women and their babies and should be the gold standard of maternity care [22]. Relationships between the woman and their care providers are also important. Karlstrom et al. [23], confirm this finding, concluding that women feel that a positive experience is generated through relationships and the woman’s own ability to focus on herself to foster control. Reducing the feelings of isolation, not being listened to, feeling frustrated and disempowered is an important component for women not only in this study but for all women. Woman centered care and continuity provides access to appropriate social networks, good communication and relationship development, whilst creating a positive care environment where women feel understood, supported, and listened to [2].

The poor experiences of maternity care for the women in this study are not unique. Our findings demonstrated that women who experienced poor continuity, and who felt they never saw the same person twice, had feelings of isolation and not being heard. This is consistent with findings from Rayment-Jones et al. [24]. When women are exposed to poor communication, lack of continuity, not being listened to and feelings of isolation this leads to poor or negative experiences.

Women in this study wanted to have quality contact with the midwives, to ensure that they themselves, and their baby, were given appropriate care. Preparation surrounding motherhood is important and therefore antenatal education is pivotal for women throughout their pregnancy [25]. The tick box and pamphlet regime described by the women in this study did not provide women with the opportunity to engage with an in-depth discussion that they felt was necessary to prepare them for their journey ahead. Downer [25] suggests that antenatal education needs to be a meaningful experience for women, to ensure preparation for their pregnancy, birth, postnatal period, and breastfeeding. This would empower women to have a better experience with their antenatal education [25].

Giving birth and being cared for in what was described as a disjointed maternity services model resulted in women feeling that their needs could not be met and that their care was very much task orientated. It is well known that medicalisation of maternity care is growing due to the increased use of interventions supported by current evidence [21]. Women in this study often did not understand the reasons for the birthing timelines and the language used by health care providers. Unfortunately, the medical management of labour overrides the natural process of birth within a hospital setting due to time constraints and potential litigation. This may contribute to poor communication around their care, such as during their birth experience and the early beginnings of learning to breastfeed [21].

Workforce shortages and quality of care impact women’s experiences. This study highlighted that women wanted to leave the hospital, believing that their care would be better at home. Consistent with Todd et al. [26], this study found that the views of most women about their maternity care journey were impacted heavily by their perceptions of and their interactions with health care providers. Many women’s negative experiences were surrounding the workforce issues such as low staffing levels, such as the busyness of the ward. It is well documented that poor staffing levels can be problematic; staffing is an operational issue that can impact the ability of staff to provide appropriate care for women and their babies. Turner et al. [27] discuss that staffing, workloads, and communication between staff and patients can impact on postnatal care delivery within the postnatal ward. Relationships between women and midwives are important. When trust is promoted through continuity, women have positive experiences across their pregnancy journey and have better outcomes relating to their birth and breastfeeding support [6,22].

The participants experienced a maternity service model, not utilised in other Australian regional areas. The maternity service model within regional Tasmania consists of two service providers providing maternity care. The public and private hospital partnerships have contributed to a fragmented model where continuity of care is hampered and limited [28]. This is complicated by poor communication, lack of continuity models, lack of support for women and poor coordination of care through the system [24]. Improving and exploring alternate models of care for women that provide consistency and continuity reduces the fragmentation within a maternity service. This is important for women’s experiences across the continuum of care, especially for women who are of poor social circumstances and those living in rural areas [6]. Therefore, fragmented maternity service models may struggle to meet the needs of woman and their families. Women should expect and receive high-quality maternity care that is effective, safe, and equitable which benefits and empowers the woman for this major life event [9].

Overall, women cited negative experiences surrounding their maternity care experience such as feelings of isolation, conflicting advice, feeling ignored, and minimal to no continuity in contrast to the euphoric feelings of birth, immense support, guidance, and encouragement. It is important to promote appropriate models of care to meet the needs of women. Women and their families need to be active partners in care provision and involved with the service delivery and implementation of quality maternity services. Listening to and designing service models based on the women and their families’ needs is crucial to improve their experiences, especially for women in rural/remote areas [3].

### 4.1. Limitations

There were some limitations to this study. A more diverse sample, including women from other regional and rural areas, ages, and social demographic is recommended. This will help to explore the variations in the experience of care in regional areas. Another limitation of this study was that staff within this service model were not interviewed. Further research examining the perspectives of important key stakeholders such as health care and executive members of staff could be valuable.

### 4.2. Recommendations from This Research

Further exploration into alternate models of care for women is recommended for the benefit of women living in regional areas in Australia. Exploring how to provide consistency of information, reduce feelings of isolation, and promote positive relationships between women and carers is needed. Further education is required surrounding the Baby Friendly Health Initiative [29] principles, that is to support, promote and protect breastfeeding. Adherence to these principles needs to occur to ensure safe and quality care and ensure sharing of information between the patient and healthcare staff.

Care pathways are required for women experiencing postnatal care complications such as anxiety, depression, and breastfeeding difficulties to ensure effective and continuous care over the maternity care journey and to ensure a smooth transition between health service providers [30,31].

## 5. Conclusions

Women and their families are the epicentre of the maternity service. Regional women’s experiences of maternity care may be improved if health services work towards place-based continuity of care models. These models should be informed by the local women’s experiences and needs to achieve better communication, reduce feelings of isolation and more positive breastfeeding experiences.

## Data Availability

Due to the subjective and potentially identifiable nature of the data no data is available.
